# l-Arginine Alleviates LPS-Induced Oxidative Stress and Apoptosis via Activating SIRT1-AKT-Nrf2 and SIRT1-FOXO3a Signaling Pathways in C2C12 Myotube Cells

**DOI:** 10.3390/antiox10121957

**Published:** 2021-12-07

**Authors:** Ye Zhao, Qin Jiang, Xuefei Zhang, Xiaoxiao Zhu, Xia Dong, Linyuan Shen, Shunhua Zhang, Lili Niu, Lei Chen, Ming Zhang, Jun Jiang, Daiwen Chen, Li Zhu

**Affiliations:** 1 College of Animal Science and Technology, Sichuan Agricultural University, Chengdu 611130, China; zhye@sicau.edu.cn (Y.Z.); 13718@sicau.edu.cn (Q.J.); 14012@sicau.edu.cn (X.Z.); 2020202059@stu.sicau.edu.cn (X.Z.); 2020202058@stu.sicau.edu.cn (X.D.); shenlinyuan@sicau.edu.cn (L.S.); 14081@sicau.edu.cn (S.Z.); niulili@sicau.edu.cn (L.N.); chenlei815918@sicau.edu.cn (L.C.); zhangming@sicau.edu.cn (M.Z.); jjun@sicau.edu.cn (J.J.); 2 Institute of Animal Nutrition, Sichuan Agricultural University, Ya’an 625014, China

**Keywords:** C2C12 myotube cell, lipopolysaccharide, l-arginine, oxidative stress, apoptosis

## Abstract

l-arginine (l-Arg) has been reported to possess a wide range of functions, including anti-inflammatory, anti-oxidative, and anti-apoptosis. However, the role of l-Arg in LPS-induced muscle injury and its potential protective mechanism has not been well elucidated. This study aimed to investigate the effects of l-Arg on the LPS-induced oxidative stress and apoptosis in differentiated C2C12 myotube cells. Our results demonstrated that myotube cells treated with 0.2 mg/mL LPS significantly decreased cell viability. l-Arg treatment significantly suppressed LPS induced ROS accumulation and cell apoptosis. Furthermore, l-Arg improved antioxidant-related enzymes’ activities; increased antioxidant ability via Akt-Nrf2 signaling pathway; maintained the mitochondrial membrane potential (MMP); and enhanced FOXO3a expression, leading to a decrease in the mitochondrial-associated apoptotic proteins. In addition, l-Arg exposure dramatically increased the mRNA and protein expressions of SIRT1. The cytoprotective effect of l-Arg was restricted by the SIRT1 inhibitor EX527, which led to an increase in ROS level, apoptosis rate, and decreased cell MMP. The results also demonstrated that EX527 treatment significantly eliminated the effect of l-Arg on LPS-induced oxidative damage and mitochondria-mediated cell apoptosis. Our findings revealed that l-Arg could be used as a potential nutraceutical in reducing muscle injury via regulating SIRT1-Akt-Nrf2 and SIRT1-FOXO3a-mitochondria apoptosis signaling pathways.

## 1. Introduction

As the most abundant tissue in vertebrates, muscle injury seriously impacts the health of the body [1]. Muscle injury has been described as one of the most important public health problems due to its elevated prevalence and decreased health-related quality of life. At the same time, as the main nutritional organ, muscle also has a variety of functions, such as body maintenance, metabolism, and disease resistance, in response to the invasion of environmental pathogens [2]. Pathogenic bacteria infections have been known to cause muscle injury, which can trigger septic shock and result in a reduction in muscle mass [3,4]. As the main pathogenic factor of Gram-negative bacteria, lipopolysaccharide (LPS) could trigger innate immunity and cause damage [5]. Emerging studies have revealed that the cytotoxicity of LPS can induce oxidative damage and cell apoptosis in mammals [6,7]. Therefore, identification of the molecular mechanisms mediating LPS and exploration of the effective and safe strategies that attenuated LPS-induced oxidative stress and apoptosis might be beneficial for the prevention and therapy of muscle injury.

Oxidative stress is the imbalance between the reactive oxygen species (ROS) and the antioxidant defense system [8]. Two types of antioxidant defense systems, enzymatic and non-enzymatic, have been evolved in vertebrates to scavenge surplus ROS and maintain redox homeostasis [9]. Numerous studies have confirmed that the protein kinase B (Akt)- nuclear factor erythroid 2-related factor (Nrf2) signaling pathway plays an important role in regulating the expression of antioxidant genes and eventually alleviating oxidative stress [10,11]. In response to the multiple stimuli, phosphorylated Akt activates the expression of Nrf2 in the nucleus and thus improves the levels of downstream antioxidant genes [12]. In addition, as mitochondria is the mainly production site of intracellular ROS [8], which is highly susceptible to oxidative stress. Excessive ROS is also considered to be the key factor responsible for changing mitochondrial membrane permeability, resulting in mitochondrial-related apoptosis [13]. As a family of evolutionally conserved forkhead transcription factors, forkhead box protein 3 (FOXO3a) protein controls cell cycle, differentiation, oxidative stress, as well as apoptosis [14,15]. A previous study reported that phosphorylated FOXO3a alleviated senescence-induced muscle cell apoptosis by reducing mitochondrial cyt c release and cleaved Caspase-3 protein levels, implying that FOXO3a also plays an important role in the regulation of mitochondrial-related apoptosis pathway [16]. Nonetheless, the role of Akt-Nrf2 and FOXO3a pathways in regulating LPS-induced myotube cells oxidative stress and apoptosis are still unclear and insufficiently understood.

Sirtuin1 (SIRT1) is a primary NAD+ dependent deacetylase, mainly distributed in the nucleus and implicated in a variety of cellular processes, including cell survival, development, oxidative stress, aging, and apoptosis [17]. An emerging study has shown that SIRT1 activates the Akt-Nrf2 signaling pathway to alleviate AlCl3-induced neurotoxicity [9]. Ma et al. found that the knockout of SIRT1 in mouse oocytes results in the decreased expression of Nrf2 and aggravates the oxidative stress caused by aging [18]. Additionally, SIRT1 also plays a pivotal role in alleviating apoptosis. A recent study indicated that SIRT1 upregulation alleviated manganese-induced neuronal apoptosis through activation of FOXO3a [19]. Duan et al. found that Aralia Taibaitalia could facilitate the deacetylation and phosphorylation of FOXO3a by activating the expression of SIRT1, thus alleviating the apoptosis of mouse hippocampal neurons (HT22) cells [20]. Therefore, SIRT1 induction is a feasible approach for activating Akt-Nrf2 and FOXO3a pathways with certain specific stimulation. However, the role that SIRT1 plays in the regulation of the toxicological effect of a given LPS to myotube cells is so far not clearly understood.

As a conditionally essential amino acid, l-arginine (l-Arg) exerts an essential role in a wide range of physiological and pathological functions, including growth regulation, inflammation response, oxidative stress, and apoptosis [21,22,23,24]. Accumulated evidence has focused on the role of l-Arg in alleviating damage in intestinal epithelial cells [23,25], Leydig cells [26], and endometrial cells [27]. Studies in mice and vascular endothelial cell have also shown that l-Arg promotes SIRT1 expression [28,29]. Nevertheless, whether or not l-Arg exerts the protective effects against LPS-induced oxidative stress and apoptosis via SIRT1 remain unclear. Therefore, the objective of the present study was to investigate the potential protective mechanisms of l-Arg against LPS-induced oxidative stress and mitochondria-related apoptosis in myotube cells. The current study will provide new insights into the comprehensive utilization of l-Arg in the prevention and management of muscle disorders.

## 2. Materials and Methods

### 2.1. Cell Culture and Treatment

The C2C12 cell line (ATCC, Manassas, VA, USA) from the 7 to 10 passages were grown in DMEM high-glucose medium supplemented with 10% fetal bovine serum and 1% antibiotics (Gibco, Waltham, MA, USA), under a humidified atmosphere of 5% CO_2_ at 37 °C. Medium was changed every other day. At confluence about 70–80%, the medium was changed to the same amount of differentiation medium, consisting of DMEM containing 2% horse serum to induce differentiation. The differentiation medium was changed to another day for 5 days. Then the cells were starved for 6 h in the l-Arg-free differentiation medium. After that, the medium was carefully removed, and cells were washed twice with cold PBS and cultured in fresh l-Arg-free differentiation medium.

### 2.2. Cell Viability

A total of 100 µL C2C12 myoblast suspension (5 × 10^4^ cells/mL) were seeded to the wells of 96-well plates. After the cell density reached 70–80%, the culture medium was removed and the cells were washed twice with cold PBS. Then the cells were incubated with differentiation medium for 5 days to induce differentiation. On day 6, the medium was changed to l-Arg- and serum-free medium for 6 h. Cells were treated with a series of concentrations of LPS (0, 0.05, 0.1, 0.2, 0.4, 0.8 mg/mL) and/or l-Arg (0, 0.5, 2.5, 5, 15, 30 mM) for another 24 h. The cell viability was be measured according to the protocol of the CCK-8 commercial kit. The experimental cell survival was calculated by the percentage of control.

### 2.3. Intracellular ROS

The C2C12 myoblasts were cultured in 96-well plates. After the 5-day differentiation, myotube cells were treated with the LPS and/or l-Arg for 24 h. After removing the medium and washing twice with cold PBS, cells were cultured with serum-free DMEM medium with the concentration of 7 μM 6-carboxy-2′,7′-dichlorofluorescin diacetate (Molecular Probes-Invitrogen Co., Carlsbad, CA, USA) at 37 °C under dark condition for 20 min. The fluorescence (excitation/emission at 485 nm/525 nm), reflecting the ROS concentration, was measured using a fluorescence microscope (IX73, Olympus Corporation, Tokyo, Japan). The ROS level was represented as the percentage of fluorescence intensity relative to the control.

### 2.4. Measurement of Antioxidant-Related Enzyme Activities

The total antioxidant capacity (T-AOC), total superoxide dismutase (T-SOD), catalase (CAT), glutathione peroxidase (GSH-px) activities, and malondialdehyde (MDA) content were analyzed in the C2C12 myotube cells using corresponding commercial kits (T-AOC, A015-2-1; T-SOD, A001-1; CAT, A007-1-1; GSH-px, A005-1; MDA, A003-4-1; Nanjing Jiancheng Bioengineering institute, Jiangsu, China) according to the manufacturer’s instruction.

### 2.5. JC-1 Staining

The mitochondrial membrane potential was determined in the C2C12 myotube cells with JC-1 kits (Beyotime, Shanghai, China). After PBS washing, the cells were stained with JC-1 for 20 min at 37 °C. The fluorescences were detected using a fluorescence microscope. Then, the regions were randomly selected from each group and the relative fluorescence intensity of cells were measured by the software Image J.

### 2.6. Determination of Cell Apoptosis

The C2C12 cells were seeded in 6-well plates and cultured for 5 days in differentiation medium. Cell apoptosis was assessed by Annexin V-FITC and propidium iodide (PI) double staining kit (Biolegend, San Diego, CA, USA). Briefly, C2C12 myotubes from different treatments were harvested and washed two times with cold PBS. Afterwards, cells were resuspended followed by the addition of binding buffer (100 μL), and stained with Annexin V (2 μL) and PI (1 μL) for 20 min on ice. Finally, cell apoptosis was determined by flow cytometry (BD Biosciences, San Jose, CA, USA).

### 2.7. Quantitative Real-Time PCR (qRT-PCR)

The total RNA in C2C12 myotubes was isolated using RNAiso reagent (Invitrogen, Carlsbad, CA, USA). Subsequently, the 2 μg of total RNA was used to transcribe into cDNA with a reverse transcription kit (Takara, Dalian, China). The integrity and purity of the obtained RNA were assessed using denaturing gel electrophoresis and a Nano Drop^®^ 2000 spectrophotometer (Thermo Scientific, Wilmington, DE, USA). Primers for qRT-PCR in this experiment are displayed in Table 1. The qRT-PCR was performed in the CFX96 RT-PCR Detection System (Bio-Rad, Hercules, CA, USA). The β-actin was regarded as an internal control. Relative mRNA abundances were computed by the 2^−ΔΔCT^ method.

### 2.8. Nuclear and Cytoplasmic Extraction

The cytoplasmic and nuclear protein fractions were extracted from C2C12 myotubes using a nuclear and cytoplasmic protein extraction kit (Beyotime, Shanghai, China) according to the manufacturer’s instructions. In brief, the lysates were ultracentrifuged at 12,000× *g* for 10 min at 4 °C, and the supernatants were collected as the cytoplasmic fraction. The pelleted nuclei were resuspended in a buffer containing 1 mM PMSF. After 30 min at 4 °C, lysates were centrifuged, and supernatants containing the nuclear proteins were stored at −80 °C. The concentration of protein was measured by BCA assay (Beyotime, Shanghai, China).

### 2.9. Western Blotting

Protein from C2C12 myotubes was isolated using RIPA lysis with 1 mM phenylmethanesulfonyl fluoride (Amresco, OH, Solon, USA) and proteinase inhibitors (Beyotime, Shanghai, China) on ice. The protein contents were quantified by BCA assay (Beyotime, Shanghai, China). Afterwards, the supernatant (20 μg of total protein) was loaded onto the polyacrylamide gel and run 125 V for 2 h and transferred to PVDF membrane. The membranes were blocked and then exposed to primary antibodies (SIRT1, Cell Signaling, 1:1000, 9475T; Akt, Cell Signaling, 1:1000, 4691S; phospho-Akt (p-Akt), Cell Signaling, 1:2000, 4060S; FOXO3a, ZenBio, 1:1000, 380728; phospho-FOXO3a (p-FOXO3a), Abcam, 1:1000, ab154786; Nrf2, ZenBio, 1:1000, 340675; Keap1, ZenBio, 1:1000, R26935; Bcl-2, Santa Cruz, 1:500, sc7382; BAX, Santa Cruz, 1:500, sc7480; Caspase-9, Cell Signaling, 1:1000, 9502T; Caspase-3, Cell Signaling, 1:1000, 14220T; LaminB1, ZenBio, 1:1000, 384825; β-actin, Cell Signaling, 1:1000, D6A8) overnight at 4 °C. Next, the membranes were washed with TBST four times and then subjected to the corresponding secondary antibody (HRP-conjugated) at 25 °C for 2 h. Then, bands were visualized by ECL chemiluminescence kit. The β-actin or LaminB1 was used as a sample loading control. The protein densitometry was analyzed by the Gel-Pro Analyzer.

### 2.10. Statistical Analysis

All data are presented as mean ± SEM. The statistical analysis was performed by SPSS 20.0 (SPSS Inc., Chicago, IL, USA). Data were compared between different groups by two-tailed Student’s *t*-test and/or one-way analysis of variance (ANOVA) followed by Tukey’s post hoc tests. *p*-value < 0.05 and <0.01 were considered to determine the significance level.

## 3. Results

### 3.1. l-Arg Attenuated LPS-Mediated Cytotoxicity in Myotube Cells

**Figure 1:**
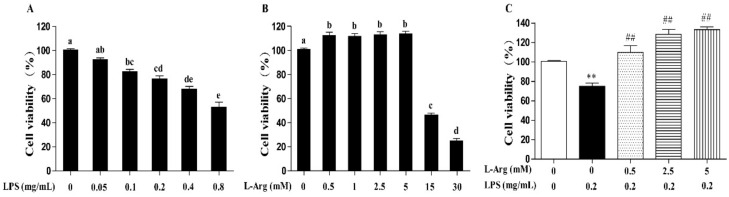
l-Arg ameliorated LPS-induced cytotoxicity in mice C2C12 myotube cells. Cell viability was detected using a CCK-8 assay. (**A**) C2C12 myotube cells were cultured with 0, 0.05, 0.1, 0.2, 0.4, 0.8 mg/mL LPS for 24 h. (**B**) The C2C12 myotube cells were treated with different concentrations of l-Arg (0, 0.5, 1, 2.5, 5, 15, and 30 mM) for 24 h. (**C**) C2C12 myotube cells were incubated with 0, 0.5, 2.5, or 5 mM l-Arg and 0.2 mg/mL LPS for 24 h. Data are presented as mean ± SEM of at least six independent experiments, different letter (a, b, c, d, and e) in A and B denotes significant difference (*p* < 0.05), ** *p* < 0.01, significantly different from control cells (l-Arg (-) and LPS (-)); ^##^ *p* < 0.01, significantly differently from cells treated with LPS only.

Incubation with cells for 24 h revealed that LPS induced cell death in a concentration-dependent manner, with 0.1 mg/mL and higher concentrations causing significant inhibition (*p* < 0.05) (Figure 1A). The 0.2 mg/mL LPS was chosen for subsequent experiments. As presented in Figure 1B, the myotube cells were treated with a series of concentrations (0.5, 1, 2.5, 5, 15, and 30 mM) l-Arg for 24 h. The results showed that compared with l-Arg-free group, treated with 0.5–5 mM of l-Arg increased the cell viability, while exposure to 15 and 30 mM l-Arg for 24 h decreased the cell viability. Then the myotube cells were pretreated with 0.5, 2.5, and 5 mM l-Arg for 1 h and 0.2 mg/mL LPS for an additional 24 h. These concentrations of 2.5 and 5 mM l-Arg significantly attenuated the decreased cell vitality caused by LPS (Figure 1C).

### 3.2. l-Arg Mitigated LPS-Induced Oxidative Stress in Myotube Cells

**Figure 2:**
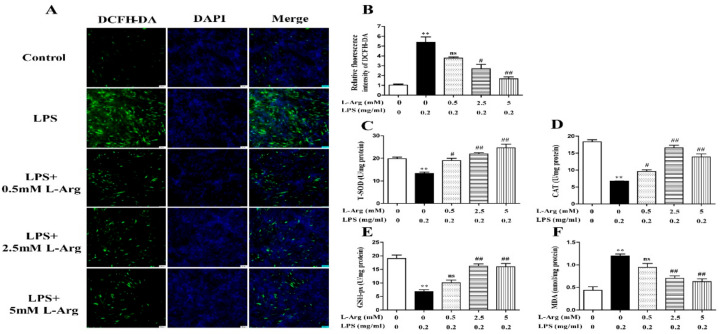
l-Arg reduced LPS-induced oxidative stress in mice C2C12 myotube cells. The myotube cells were treated with and without 0.2 mg/mL LPS in the absence or presence of 0.5, 2.5, and 5 mM l-Arg for 24 h. (**A**) Representative microphotographs showing intracellular ROS content. (**B**) Relative fluorescence density of DCFH-DA. (**C**–**F**) T-SOD, CAT, and GSH-px activities and MDA content in myotube cells. The data represent the mean ± SEM of at least three independent experiments, ** *p* < 0.01, significantly different from control cells (l-Arg (-) and LPS (-)); ^#^ *p* < 0.05, ^##^ *p* < 0.01, significantly differently from cells treated with LPS only; ns, no significantly; scale bar: 50 μm.

To determine whether l-Arg could exert a protective effect against LPS-induced oxidative stress, the ROS level was detected by fluorescence staining. As present in Figure 2A,B, l-Arg addition significantly inhibited the increased ROS production induced by LPS treatment in myotube cells. Furthermore, the activities of T-AOC, T-SOD, CAT, GSH-px, and the content of MDA were measured. As expectedly, l-Arg significantly increased T-AOC, T-SOD, CAT, and GSH-px activities in LPS-induced myotube cells (Figure 2C–E). In addition, l-Arg blocked the LPS-induced increase in MDA content in cells (Figure 2F).

**Figure 3:**
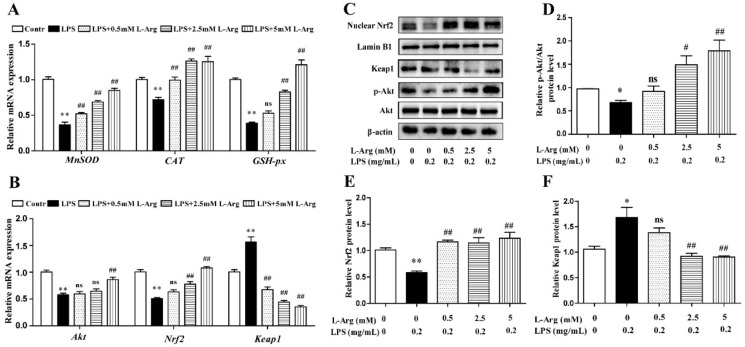
l-Arg attenuated LPS-induced oxidative stress by Akt-Nrf2 signaling pathway in mice C2C12 myotube cells. The myotube cells were treated with and without 0.2 mg/mL LPS in the absence or presence of 0.5, 2.5, and 5 mM l-Arg for 24 h. (**A**) The MnSOD, CAT, and GSH-px mRNA levels were detected by RT-PCR. (**B**) The Akt, Nrf2, and Keap1 mRNA levels were also detected using RT-PCR. (**C**) Representative Western blot images of p-Akt, Akt, Nrf2, and Keap1 in the cells. (**D**–**F**) The protein expression and quantitation of p-Akt, Akt, Nrf2, and Keap1 in the nucleus and cytoplasm. All data represent the mean ± SEM of at least three independent experiments, * *p* < 0.05, ** *p*< 0.01, significantly different from control cells (l-Arg (-) and LPS (-)); ^#^ *p*< 0.05, ^##^ *p*< 0.01, significantly differently from cells treated with LPS only; ns, no significantly.

To further confirm the mechanism by which l-Arg acted against LPS-induced oxidative stress, the expression of antioxidant-related genes and proteins were measured using RT-PCR and Western blot analysis. As shown in Figure 3A,B, l-Arg treatment significantly inhibited the Keap1 mRNA level and increased the mRNA levels of MnSOD, CAT, GSH-px, Nrf2, and Akt. Western blot analysis revealed that LPS incubation reduced the protein ratio of p-Akt/Akt (Figure 3C,D) and the nuclear protein level of Nrf2 (Figure 3C,E), which were significantly reversed by 2.5 and 5 mM l-Arg treatment. The protein expression of Keap1 showed an opposite trend to that of Nrf2 (Figure 3C,F). 

### 3.3. l-Arg Mitigated LPS-Induced Apoptosis in Myotube Cells

**Figure 4:**
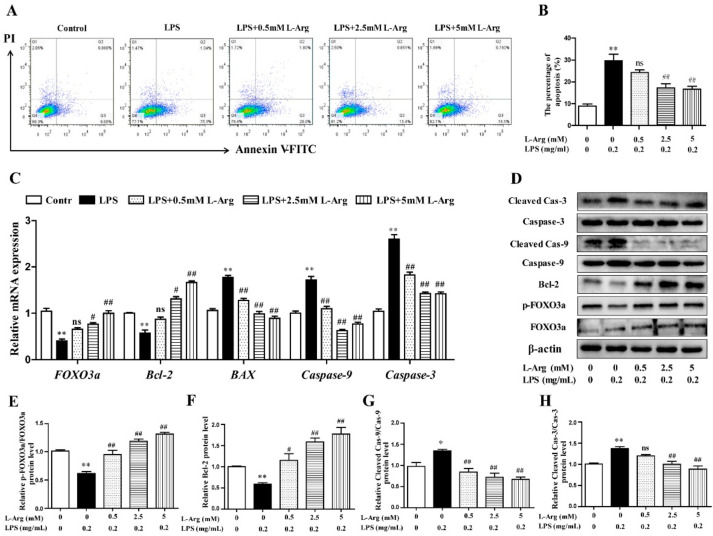
l-Arg suppressed LPS-induced apoptosis in mice C2C12 myotube cells. The myotube cells were pretreated with l-Arg (0, 0.5, 2.5, 5 mM) for 1 h and LPS (0.2 mg/mL) was added for an additional 24 h. (**A**) The apoptosis of cells was detected by flow cytometry analysis. (**B**) The percentages of apoptotic cells were counted. (**C**) RT-PCR was performed to detect the mRNA levels of FOXO3a, Bcl-2, BAX, Caspase-9, and Caspase-3. (**D**) Western blot analysis confirmed the expressions of FOXO3a, Bcl-2, Caspase-9, and Caspase-3. (**E**–**H**) The bar graph showed the quantification of p-FOXO3a/FOXO3a, Bcl-2, Cleaved-Caspase-9/Caspase-9, and Cleaved-Caspase-3/Caspase-3, respectively. The data represent the mean ± SEM of at least three independent experiments, * *p*< 0.05, ** *p*< 0.01, significantly different from control cells (l-Arg (-) and LPS (-)); ^#^ *p*< 0.05, ^##^ *p*< 0.01, significantly differently from cells treated with LPS only; ns, no significantly.

To characterize apoptotic profiles induced by LPS, myotube cells were incubated with 0.5, 2.5, and 5 mM l-Arg for 1 h, followed by incubation with 0.2 mg/mL LPS for 24 h. As the results presented in Figure 4A,B, flow cytometry analysis revealed that the percentage of cells with apoptotic features in the LPS-treated group was visibly higher than that of the control. l-Arg addition significantly decreased the percentage of apoptotic cells after LPS treatment.

To further demonstrate the possible mechanism of l-Arg in anti-apoptosis underlying LPS-induced cells, the anti-apoptosis related genes and proteins were measured using RT-PCR and Western blot analysis. As shown in Figure 4C, l-Arg addition dramatically increased FOXO3a and Bcl-2 mRNA levels. In contrast, l-Arg administration significantly decreased the mRNA expressions of BAX, Caspase-9, and Caspase-3. Western blot analysis indicated that the decreased protein levels of p-FOXO3a/FOXO3a and Bcl-2 in LPS-induced cells were markedly increased by l-Arg administration (Figure 4D–F). Moreover, the increased protein levels of cleaved Caspase-9/3 in LPS-induced myotubes cells were dramatically reversed by l-Arg treatment (Figure 4D,G,H). 

### 3.4. l-Arg Increased the Expression Levels of SIRT1 in Myotube Cells

**Figure 5:**
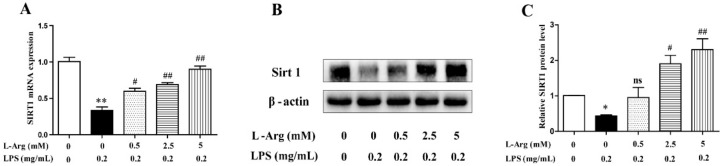
l-Arg increased the expression of SIRT1 in mice C2C12 myotube cells. The myotube cells were pretreated with l-Arg (0, 0.5, 2.5, 5 mM) for 1 h and LPS (0.2 mg/mL) was added for an additional 24 h. (**A**) RT-PCR was performed to detect the mRNA level of Sirt1. (**B**) Western blot analysis confirmed the expression of Sirt1. (**C**) The bar graph showed the quantification of Sirt1. The data represent the mean ± SEM of at least three independent experiments, * *p*< 0.05, ** *p*< 0.01, significantly different from control cells (l-Arg (-) and LPS (-)); ^#^ *p* < 0.05, ^##^ *p* < 0.01, significantly differently from cells treated with LPS only; ns, no significantly.

SIRT1 have been demonstrated to play a crucial role in many pathophysiological processes including oxidative stress and apoptosis in mammal [30]. So, the expression level of SIRT1 was detected by RT-PCR and Western blot. As shown in Figure 5, after administration with LPS for 24 h, the SIRT1 mRNA and protein levels were significantly decreased. However, the decrease was attenuated by l-Arg treatment. 

### 3.5. l-Arg Alleviated LPS-Induced Myotube Cells Oxidative Stress through SIRT1

**Figure 6:**
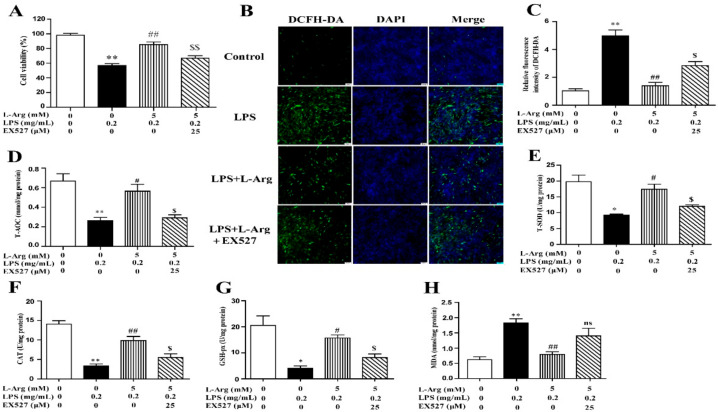
l-Arg ameliorated LPS-induced oxidative stress through SIRT1. The myotube cells were pretreated with 25 μM EX527 for 24 h, then 5 mM l-Arg was added to cell culture for 1 h prior to LPS (0.2 mg/mL) stimulation for an additional 24 h. (**A**) Cell viability was detected using a CCK-8 assay. (**B**) Cells were stained with DCFH-DA. The fluorescence intensity was tested using fluorescent microscopy (Scale bar = 50 μm). (**C**) Quantitative analysis of relative fluorescence intensity of DCFH-DA. (**D**–**H**) The MDA content and the activities of T-SOD, CAT, and GSH-px in myotube cells. Data are presented as the mean ± SEM of at least three independent experiments, * *p* < 0.05, ** *p* < 0.01 versus the Control group; ^#^ *p* < 0.05, ^##^ *p* < 0.01 versus the LPS group; ^$^ *p* < 0.05, ^$$^ *p* < 0.01 versus the LPS + l-Arg group; ns, no significantly.

To investigate the involvement of SIRT1 in the protective effect of l-Arg in LPS-explored cells, we used a specific inhibitor (EX527) to inhibit SIRT1. From the results of Western blot analysis, exposure to EX527 for 24 h significantly decreased SIRT1 protein levels. As shown in Figure 6A, 5 mM l-Arg up-regulated myotube cells viability, which could be reversed by EX527, further confirming that l-Arg alleviated LPS-induced cytotoxicity through activating SIRT1.

**Figure 7:**
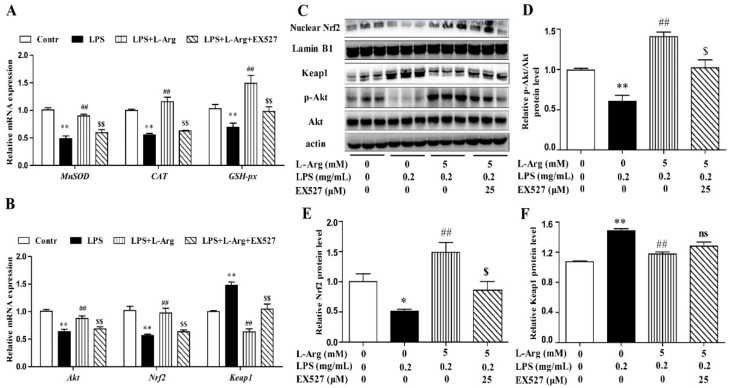
l-Arg attenuated LPS-induced oxidative stress by SIRT1-Akt-Nrf2 signaling pathway in mice C2C12 myotube cells. The C2C12 myotube cells were pretreated with 25 μM EX527 for 24 h, then 5 mM l-Arg was added to cell culture for 1 h prior to LPS (0.2 mg/mL) stimulation for an additional 24 h. (**A**) The MnSOD, CAT, and GSH-px mRNA levels were detected by RT-PCR. (**B**) The Akt, Nrf2, and Keap1 mRNA levels were also detected using RT-PCR. (**C**) Representative Western blot images of p-Akt, Akt, Nuclear Nrf2, and Keap1 in the cells. (**D**–**F**) The protein expression and quantitation of p-Akt, Akt, Nrf2, and Keap1 in the nucleus and cytoplasm. All data represent the mean ± SEM of at least three independent experiments, * *p* < 0.05, ** *p* < 0.01 versus the Control group; ^##^ *p* < 0.01 versus the LPS group; ^$^ *p* < 0.05, ^$$^ *p* < 0.01 versus the LPS + l-Arg group; ns, no significantly.

To further explore the mechanism of SIRT1 alleviating oxidative stress in LPS-induced myotube cells by l-Arg, the intracellular ROS levels were measured using a DCFH-DA probe. As shown in Figure 6B,C, the addition of 5 mM l-Arg significantly alleviated the LPS-induced ROS increase and EX527 significantly inhibited the beneficial effect of l-Arg. We also examined the effect of EX527 on antioxidant-related enzymes’ activities and genes’ expressions in myotube cells (Figure 6D–G). l-Arg increased T-AOC, T-SOD, CAT, and GSH-Px activities, which were significantly abolished by EX527. The mRNA levels of MnSOD, CAT, and GSH-px were significantly up-regulated after l-Arg and LPS treatment, while EX527 significantly inhibited the upregulation (Figure 7A). To determine whether SIRT1 alleviates LPS-induced oxidative stress by l-Arg via Akt-Nrf2 signaling pathway in myotube cells. The expressions of Akt, Nrf2, and Keap1 were detected by RT-PCR (Figure 7B) and Western blot (Figure 7C–F). What the increases in Akt and Nrf2 mRNA levels induced by l-Arg were significantly eliminated by the EX527, while the mRNA levels of Keap1 was opposite to that of Nrf2. In addition, SIRT1 inhibitors significantly abolished the inhibitory effect of l-Arg on the LPS-induced decrease in p-Akt/Akt protein levels. Meanwhile, EX527 significantly eliminated the nuclear accumulation of Nrf2 induced by l-Arg. However, l-Arg, LPS, and EX527 co-incubated cells significantly abolished the down-regulation effect of l-Arg on Keap1 protein level.

### 3.6. l-Arg Alleviated LPS-Induced Myotube Cells Apoptosis by SIRT1

**Figure 8:**
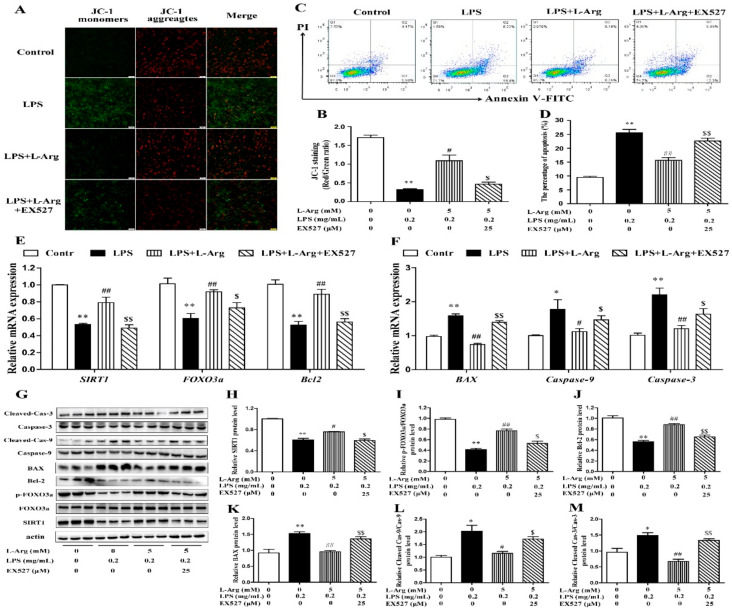
l-Arg relieved LPS-induced apoptosis via SIRT1-FOXO3a signaling pathway in mice C2C12 myotube cells. The C2C12 myotube cells were pretreated with 25 μM EX527 for 24 h, then 5 mM l-Arg was added to cell culture for 1 h prior to LPS (0.2 mg/mL) stimulation for an additional 24 h. (**A**,**B**) Mitochondrial membrane potential levels of cells were detected by JC-1 staining and the percentages of cells were counted. (**C**,**D**) Apoptosis of cells was detected by flow cytometry analysis and the percentages of apoptotic cells were counted. (**E**,**F**) RT-PCR was performed to detect the mRNA levels of SIRT1, FOXO3a, Bcl-2, BAX, Caspase-9, and Caspase-3. (**G**) Western blot analysis confirmed the expression of SIRT1, FOXO3a, Bcl-2, BAX, Caspase-9, and Caspase-3. (**H**–**M**) The bar graph showed the quantification of SIRT1, p-FOXO3a/FOXO3a, Bcl-2, BAX, Cleaved Caspase-9/Caspase-9, and Cleaved Caspase-3/Caspase-3, respectively. The data represent the mean ± SEM of at least three independent experiments, * *p* < 0.05, ** *p* < 0.01 versus the Control group, ^#^ *p* < 0.05, ^##^ *p* < 0.01 versus the LPS group, ^$^ *p* < 0.05, ^$$^ *p* < 0.01 versus the LPS + l-Arg group.

The persistently high level of intracellular ROS would cause mitochondrial dysfunction, then activate the mitochondria-related apoptosis pathway and eventually cause cell and tissue damage [8,31]. MMP levels were detected with JC-1 staining (Figure 8A). As expected, EX527 significantly abolished the effects of l-Arg on MMP in LPS-treated myotube cells (Figure 8B). Furthermore, the apoptosis cells were detected by flow cytometry (Figure 8C). The apoptosis rate of cells was shown in Figure 8D. EX527 significantly abolished the down-regulation effect of l-Arg on apoptosis rate.

In order to determine whether SIRT1 is involved in l-Arg alleviating LPS-induced apoptosis via the FOXO3a-mediated mitochondrial apoptosis signaling pathway in myotube cells, the expression levels of SIRT1, FOXO3a, BAX, Bcl-2, Caspase-9, and Caspase-3 were detected by RT-PCR (Figure 8E,F) and Western blot (Figure 8G–M). l-Arg increased the SIRT1, FOXO3a, and Bcl-2 mRNA levels, which were significantly inhibited by the EX527. In contrast, the mRNA levels of BAX, Caspase-9, and Caspase-3 were opposite to that of Bcl-2. In addition, SIRT1 inhibitors significantly abolished the inhibition of l-Arg on LPS-induced SIRT1 and p-FOXO3a/FOXO3a protein level decrease. At the same time, EX527 significantly eliminated the l-Arg-induced decrease in BAX protein levels. However, co-incubation with l-Arg, LPS, and EX527 significantly eliminated the down-regulation effects of Cleaved Caspase-9/Caspase-9 and Cleaved Caspase-3/Caspase-3 protein levels by l-Arg. 

## 4. Discussion

Over the past decades, it has been accepted that muscle could exert a spontaneous immune behavior response to external pathogen stimulation [2]. LPS is the main component of the cell wall of Gram-negative bacteria, and as the main virulence factor, it causes serious pathological reactions in animals [5,32]. However, how to effectively mitigate muscle damage caused by LPS has become a thorny issue in the farming industry. l-Arg is a semi-essential amino acid with a wide range of effects, and a variety of studies have shown that an appropriate amount of l-Arg can effectively improve the body’s immunity [33]. Oxidative stress and apoptosis are considered to be among the most effective mechanisms of animal’s immune defense [5]. Previous studies showed that l-Arg could induced C2C12 myoblasts differentiation. Therefore, in this study, we used differentiated C2C12 cells [34,35]. The different concentrations of l-Arg were used to treat LPS-induced C2C12 myotube cells, to explore the mechanism of l-Arg alleviating oxidative stress and apoptosis, and to provide a new idea for alleviating muscle injury.

Cell viability is the most direct indicator of cell growth and the level of cell viability directly reflects the survival state of cells [36]. The results of this study showed that different concentrations of LPS reduced C2C12 myotube cells viability in a dose-dependent manner. Similarly, Shang et al. also found that treatment with 0.1 mg/mL LPS for 24 h significantly reduced cell viability in C2C12 myoblast cells [37]. The decline in cell viability is usually accompanied by severe oxidative stress and apoptosis. This result also confirmed that LPS treatment for 24 h resulted in a significant increase in intracellular ROS levels and apoptosis rates. Consistent with the results of this study, the intracellular ROS level of C2C12 myoblast cells was increased by 40% after being treated with LPS for 6 h [38]. Thus, it could be concluded that LPS-induced muscle injury might be caused by increasing ROS and apoptosis levels, reducing cell viability, and ultimately leading towards cell and tissue damage. This further indicated that the LPS-induced muscle injury model was successfully constructed.

Muscle is the main organ of energy metabolism and homeostasis maintenance of the body [1,39]. During the growth and development of animals, muscle is also particularly vulnerable to invasion by pathogenic microorganisms [2]. As a result, muscle tissue is vulnerable to a high risk of oxidative stress. Excessive oxidative stress is often accompanied by excessive accumulation of ROS and MDA [40]. The present study showed that LPS significantly increased ROS levels and MDA contents in myotube cells, while l-Arg with appropriate concentration significantly reduced the levels of ROS and MDA. This was similar to studies on pig intestinal epithelial cells (IPEC-J2) [21] and mouse vascular endothelial cells [29]. Previous studies have shown that persistently high ROS levels lead to the imbalance of oxidative and antioxidant systems in the body [6,39]. Among them, antioxidant enzymes (SOD, CAT, and GSH-Px), as the main antioxidant substances, can resist excessive ROS attacks and maintain redox homeostasis [41]. The SOD could convert superoxide free radicals to H_2_O_2_, which was degraded to O_2_ by CAT and GSH-px [42]. The present study confirmed that the activities of antioxidant enzymes significantly decreased after LPS treatment, while l-Arg pretreatment significantly increased the enzymes activities. These results indicate that l-Arg effectively protected against LPS-induced oxidative stress by increasing antioxidant enzymes activities.

The activities of antioxidant enzymes are closely related to the transcriptional level of corresponding genes. In the present study, l-Arg increased MnSOD, CAT and GSH-px mRNA levels, suggesting that l-Arg increased antioxidant enzymes activities in LPS-induced myotube cells, which might be related to the corresponding mRNA levels. Similar results have been found in rat liver [33] and IPEC-J2 cells [23]. These results suggest that l-Arg preconditioning could protect myotube cells from LPS-induced oxidative stress by increasing antioxidant genes expressions. A large number of studies have shown that the Nrf2-Keap1 signaling pathway performs the function of anti-oxidative stress [43,44]. Under normal circumstances, Nrf2 binds to Keap1 and localizes in the cytoplasm, leading to ubiquitination-proteasomal degradation mediated by the E3 ubiquitin ligase complex [45]. When cells are subjected to oxidative stress, the degradation of Nrf2 is reduced, and the Nrf2 protein dissociates from Keap1, and the former enters the nucleus to activate relevant antioxidant reaction elements and exerts its antioxidant function [46,47]. In the present study, we found that LPS-induced oxidative stress in myotube cells, significantly decreased the mRNA level of Nrf2, and significantly increased the mRNA level of Keap1. Meanwhile, l-Arg also significantly promoted the expression of Nrf2 gene in LPS-induced myotube cells, and significantly down-regulated the mRNA level of Keap1. Consistent with the mRNA level, l-Arg significantly increased the protein expression level of Nrf2 in the nucleus and significantly decreased the protein expression level of Keap1 in the cytoplasm. These results suggest that activation of Nrf2 is crucial for l-Arg to alleviate LPS-induced oxidative stress in myotube cells. Previous studies have also shown that l-Arg is a potential regulator of Nrf2 in the body. After feeding the rats with l-Arg, it was found that the appropriate concentration of l-Arg could activate Nrf2 in the liver and inhibit Keap1 mRNA and protein levels [33]. Zhang et al. found that l-Arg could alleviate oxidative damage of sheep intestinal epithelial cells induced by LPS by increasing the protein expression of Nrf2 [25]. These results suggest that l-Arg reduced LPS-induced oxidative stress in myotube cells by activating the Nrf2-Keap1 signaling pathway.

Although the present study has confirmed that l-Arg activated the Nrf2 signaling pathway and improved the antioxidant capacity in myotube cells, how l-Arg regulates the nuclear translocation of Nrf2 is still unclear. Parallel studies have found that Akt activates the expression of Nrf2 to inhibit oxidative stress [10,12,48]. In addition, phosphorylated Akt enhances Nrf2 nuclear translocation and protects against AlCl3-mediated oxidative stress in PC12 cells [9]. Resveratrol can protect IPEC-J2 cells from H2O2-induced oxidative stress through the Akt-Nrf2 signaling pathway [49]. Our results showed that LPS inhibited the activation of Akt, while l-Arg increased the phosphorylation level of Akt, thereby activating the Akt signaling pathway in myotube cells. Barbosa et al. found that l-Arg increased the phosphorylation level of Akt in a NO-dependent manner, thereby enhancing glucose and lipid metabolism in rat L6 myotubule cells [22]. According to these results, l-Arg enhanced the expression of antioxidant genes by activating an Akt-Nrf2 signaling pathway and alleviating the LPS-induced oxidative damage in myotube cells.

Under physiological and pathological conditions, ROS and mitochondria play an important role in the process of apoptosis [13,31,50]. Mitochondria is the main site of ROS production and an important target of ROS [8]. Previous studies have shown that excessive ROS-induced oxidative stress, led to change in mitochondrial outer membrane permeability and the MMP, and eventually induced cell apoptosis in a mitochondria-dependent manner [8,51]. The permeability of the mitochondrial outer membrane is associated with the recombination of the Bcl-2 protein family [52]. When subjected to external stimulation, the binding of BAX, originally distributed in cytoplasm, and the BH3 binding domain distributed in mitochondrial Bcl-2, is altered, leading to the accumulation of BAX protein in the outer membrane of mitochondria and the change in MMP [53,54]. The altered mitochondrial membrane permeability is accompanied by the transformation of MMP, inducing Caspase cascade activation and eventually leading to cell apoptosis [55]. The present results show that LPS significantly increases the apoptosis rates of myotube cells, while l-Arg significantly decreases the apoptosis rates of myotube cells. Consistent with previous studies, LPS induced the apoptosis of rat adipocytes [56], mouse osteoclasts [57], and mouse kidney cells [7] by activating mitochondria-related apoptosis signaling pathways. However, l-Arg has been reported to inhibit apoptosis in mouse Leydig cells [26], human endometrial cells [27], and sheep intestinal epithelial cells [58]. In this study, we found that LPS down-regulated MMP, and l-Arg treatment significantly reversed this phenomenon. These results suggest that l-Arg alleviated LPS-induced apoptosis by changing the MMP of myotube cells. Furthermore, the mRNA levels of BAX, Caspase-9, and Caspase-3 were significantly up-regulated, while Bcl-2 mRNA levels were significantly down-regulated in the LPS-treated group. Consistent with transcription levels, LPS treatment also significantly increased cleaved Caspase-9/3 protein expressions and down-regulated Bcl-2 protein expression. l-Arg treatment significantly reversed the changes in mRNA and protein levels of mitochondrial apoptosis-related genes induced by LPS. Similar results were found in mouse lung tissue [59] and sheep epithelial cells [25]. From these results and the present study, it was concluded that l-Arg alleviated LPS-induced apoptosis by regulating the mRNA and protein levels of mitochondria-related apoptosis pathway in myotube cells.

Although the current and previous studies have found that l-Arg can regulate mitochondria-related apoptosis pathways to alleviate apoptosis, further studies are also needed to ascertain how l-Arg mediates the expression of related genes and how it affects mitochondrial pathways. FOXO protein is an evolutionally-conserved transcription factor family with functions of controlling cell cycle, differentiation, and resistance to oxidative stress and apoptosis [60]. A large amount of evidence has shown that FOXO3a not only plays an important role in the regulation of skeletal muscle atrophy [61,62], but also plays a key role in autophagy and apoptosis [16]. In general, FOXO3a is the downstream of Akt and controlled by Akt phosphorylation, leading to nuclear transfer [14]. FOXO3a resides in the nucleus and controls the transcription of target genes in the absence of external stress and Akt activation. However, in response to external stimulation and Akt activation, FOXO3a is activated by phosphorylation at Thr32, Ser253, and Ser315, leading to extracellular migration and inhibition of transcription [63]. In mice muscle with chronic resistance to exercise, phosphorylated activated FOXO3a was found to alleviate senescence-induced apoptosis of muscle cells by reducing mitochondrial Cyt C release and cleaved Caspase-3 protein levels [16]. In this study, it was found that FOXO3a mRNA level and the ratio of p-FOXO3a/FOXO3a were significantly down-regulated in the LPS-treated group, while FOXO3a mRNA and p-FOXO3a protein levels were up-regulated in a dose-dependent manner after l-Arg supplementation. These results indicated that l-Arg significantly alleviated LPS-induced inactivation of FOXO3a signaling pathway and reduced the occurrence of cell apoptosis. The positive effects of l-Arg on the activation of FOXO3a signaling pathway may be partly ascribed to a metabolite of Arg. Fan et al. reported that spermidine, a metabolite of Arg, could activate the FOXO3a signaling pathway and alleviate the apoptosis of skeletal muscle induced by type D Galactose in rats [15]. These results indicate that administering l-Arg exerts a protective effect against LPS-induced apoptosis via the FOXO3a-mitochondrial apoptosis signaling pathway in myotube cells. However, how l-Arg regulates FOXO3a to alleviate the activation of mitochondrial apoptosis-related signaling pathways is not particularly clear at present, and further studies are needed.

SIRT1 is a component of class III histone deacetylases, which has been reported to be involved in a variety of biological processes, and also plays an important role in oxidative stress and apoptosis [30,64]. The present study showed that the mRNA and protein levels of SIRT1 were significantly down-regulated in the LPS group. However, with the increase in l-Arg concentration, the mRNA and protein levels of SIRT1 increased in a dose-dependent manner. Studies have shown that LPS treatment significantly down-regulated the expression of SIRT1 in PC12 cells [65], RAW264.7 cells [66], and mouse myocardium [67]. Similar to the results of this study, l-Arg up-regulated the expression of SIRT1 in human vascular endothelial cells [29]. Chen et al. also found in C2C12 cells that l-Arg could promote the transformation of muscle fibers by increasing the SIRT1 protein level [28]. These results suggest that l-Arg and SIRT1 might interact with each other in LPS-induced oxidative stress and apoptosis, thereby enhancing SIRT1-mediated downstream biological functions.

Studies have confirmed that EX527 can significantly inhibit the expression level of SIRT1 in different cells and tissues [68,69]. Therefore, we selected EX527 to further investigate whether l-Arg could inhibit LPS-induced cell damage by regulating oxidative stress via SIRT1 in myotube cells. The results of this study show that compared with LPS+l-Arg group, ROS levels were significantly up-regulated after pretreatment with EX527 in myotube cells. Similarly, Zhao et al. found that EX527 significantly increased the oxidative stress of the cerebral cortical nerve cells in mice [68]. Another study showed that treatment with EX527 significantly blocked melatonin-alleviated oxidative stress in mouse testicular stromal cells [64]. Notably, this study found that the l-Arg-mediated high phosphorylation level of Akt and high expression of Nrf2 in the nucleus, as well as the expression of downstream antioxidant genes, were significantly inhibited by EX527. Similarly, the EX527 group significantly abolished the beneficial effects of resveratrol on alleviating Nrf2 protein levels and the activities of SOD, CAT, and GSH-Px in rats myocardial ischemia-reperfusion injury [70]. However, Yang et al. found that treatment with EX527 significantly reduced the activation of Nrf2 in the kidney of diabetic rats, but had no effect on the phosphorylation level of Akt [71]. Lu et al. found that DiDang Tang alleviated AlCl3-induced oxidative damage in PC12 cells by activating SIRT1-mediated Akt-Nrf2 signaling pathway [9]. Existing evidence indicated that there was an interaction between SIRT1 and Akt. Previous studies have shown that SIRT1 can increase the membrane localization and activation of Akt by deacetylation of Akt [72], and can also activate the Akt signaling pathway by interacting with the upstream PTEN of Akt [73]. These results indicate that l-Arg mediated the Akt-Nrf2 signaling pathway in a SIRT1-dependent manner. Even so, how l-Arg alleviates oxidative stress by regulating SIRT1 and Akt-Nrf2 signaling pathway remains to be further studied.

Previous reports and our present study have found that reducing the expression of SIRT1 significantly increases ROS levels, where the accumulation of ROS in cells changes mitochondrial permeability, impacts the normal membrane potential level, and eventually triggers mitochondrial apoptosis pathway [74,75]. In order to understand the role of SIRT1 in l-Arg-alleviated myotube cell apoptosis, we further treated the cells with EX527 in combination with l-Arg. In the present study, EX527 significantly reversed the l-Arg-induced increase in MMP and decrease in apoptosis rate of myotube cells. Consistent with the results of this study, in a model of alveolar epithelial cell injury induced by cigarette smoke, it was found that treatment with NaHS increased the level of MMP and decreased the rates of cell apoptosis. However, inhibition of SIRT1 weakens the protective effects of NaHS [76]. Tian et al. found that EX527 significantly increased the low level of mitochondrial membrane potential and apoptosis of cardiomyocytes induced by ethanol [69]. Interestingly, mRNA levels and protein levels of genes in mitochondrial apoptosis-related pathways corresponding to MMP levels and apoptosis rates were also affected by EX527 treatment, which significantly reversed the effect of l-Arg. This is parallel to the fact that EX527 treated A459 cells can aggravate the changes in mitochondrial apoptosis pathway genes induced by cigarette smoke [76]. These results suggest that l-Arg can regulate the mitochondrial apoptosis-related signaling pathway by regulating the expression of SIRT1, and thus alleviate LPS-induced apoptosis in myotube cells.

## 5. Conclusions

In summary, this study first demonstrated that l-Arg and SIRT1 alleviated LPS-induced oxidative stress and apoptosis in C2C12 myotube cells. Moreover, l-Arg was found as a potent molecule that rescued the myotube cells from LPS-induced oxidative stress and apoptosis by regulating SIRT1-Akt-Nrf2 and SIRT1-FOXO3a-mitochondrial apoptosis-related pathways.

## Figures and Tables

**Table 1:** The primer sequences used for RT-qPCR.

Gene Name	Sequence (5′-3′)	TM (°C)
SIRT1	QF: AGGGAACCTTTGCCTCATCTA	61.4
	QR: ATTGTTGTTTGTTGCTTGGTCTAC	
Akt	QF: TACTCATTCCAGACCCACGACC	60.4
	QR: GCAAGTAGTCCAGGGCAGACAC	
FOXO3a	QF: TGGATGCGTGGACCGACTT	61.4
	QR: CCAGCCCATCATTCAGATTCAT	
Nrf2	QF: TTTCAACCCGAAGCACGC	56.9
	QR: TTTCACATTGGGATTCACGC	
Keap1	QF: TGCCCCTGTGGTCAAAGTG	59.4
	QR: GGTTCGGTTACCGTCCTGC	
MnSOD	QF: ACAATCTCAACGCCACCGA	60.3
	QR: CCAGCCTGAACCTTGGACTC	
CAT	QF: CACTGACGAGATGGCACACT	59.4
	QR: TGTGGAGAATCGAACGGCAA	
GSH-px	QF: CAGGAGAATGGCAAGAATGAAG	56.9
	QR: GGAAGGTAAAGAGCGGGTGA	
Bcl-2	QF: AACCCAATGCCCGCTGT	60.4
	QR: CCTGAAGAGTTCCTCCACCAC	
BAX	QF: TGCTACAGGGTTTCATCCAGG	58.4
	QR: TGCTGTCCAGTTCATCTCCAAT	
Caspase-9	QF: CCTTCCCAGGTTTTGTCTCC	60.4
	QR: GCTTGTAAGTCCCTTTCGCAG	
Caspase-3	QF: TGACTGGAAAGCCGAAACTCT	60.4
	QR: GGGACTGGATGAACCACGAC	
β-actin	QF: GATGGTGGGAATGGGTCAGA	59.0
	QR: TCAATGGGGTACTTCAGGGTC	

## Data Availability

The data presented in this study are available in this manuscript.
